# Improving Equity of Care for Patients with Limited English Proficiency Using Quality Improvement Methodology

**DOI:** 10.1097/pq9.0000000000000486

**Published:** 2021-12-15

**Authors:** Elizabeth M. Martinez, Daniel Timothy Carr, Paul C. Mullan, Lakisha E. Rogers, Wendy L. Howlett-Holley, Coleman A. McGehee, Christopher D. Mangum, Sandip A. Godambe

## Abstract

Disparate clinical outcomes have been reported for patients with Limited English Proficiency (LEP) in the emergency department setting, including increased length of stay, diagnostic error rates, readmission rates, and dissatisfaction. Our emergency department had no standard processes for LEP patient identification or interpreter encounter documentation and a higher rate of 48-hour LEP return visits (RV) than English proficient patients. The aim was to eliminate gaps by increasing appropriate interpreter use and documentation (AIUD) for Spanish-speaking LEP (LEP-SS) patients from 35.7% baseline (10/17-05/18) to 100% by October 2020.

**Methods:**

LEP-SS patient data were reviewed in the electronic medical record to determine the AIUD and RV rates. Using the Model for Improvement and multiple Plan-Do-Study-Act (PDSA) cycles, a multi-disciplinary team encouraged stakeholder engagement and identified improvement opportunities, implemented an electronic tracking board LEP icon (PDSA1), standardized documentation using an LEP Form linked to the icon (PDSA2), and included color changes to the icon for team situational awareness (PDSA3).

**Results:**

The mean of LEP-SS patients with AIUD improved from 35.7% to 64.5% without significant changes in balancing measures. During the postintervention period (6/1/2018-10/31/2020), no special cause variation was noted from the baseline 48-hour emergency department RV rates for LEP patients (3.1%) or English proficient patients (2.6%).

**Conclusions:**

While the RV rate was not affected, this project is part of a multi-faceted approach aiming to positively impact this outcome measure. Significant improvements in AIUD were achieved without affecting balancing measures.

## INTRODUCTION

Limited English proficiency (LEP) families often receive a lower quality of care despite federal regulations such as Title VI of the Civil Rights Act of 1964, which protects patients and families from discrimination and requires organizations that receive federal funding to “take reasonable steps to provide meaningful access to each individual with LEP”.^1–3^ According to the US Census Bureau, the Hispanic/Latino population made up 18% of the total US population between 2008 and 2018 and remains the largest LEP population, where 62% reported speaking Spanish.^4,5^ Of the local LEP population, 4.0% were Spanish-speaking.^6^

Patients with LEP in the Emergency Department (ED) are frequently misidentified, either by staff or families, leading to inappropriate or no provision of language services.^7^ Due to language barriers, this population is also more likely to experience safety events, medication errors, increased hospital length of stay (LOS), unanticipated return visits to the ED, or provider encounter dissatisfaction.^8–14^ One study identified 2 target areas for ED RV improvement: early LEP identification and interpreter use.^14^ Professional interpreter use is associated with improved quality of care, including improved quality of communication, diagnostic accuracy, and sensitivity to cultural differences.^1,15,16^

In our ED, the rate of 48-hour ED return visits (RV) for LEP patients was higher than that of English proficient (EP) patients, where 79.2% of LEP RV returned for the same complaint. Additionally, processes to identify LEP patients who needed an interpreter and measure interpreter use were either missing or not standardized. This project’s global aim was to eliminate this LEP/EP RV gap and improve the quality of care for the LEP population. By ensuring appropriate interpreter use and documentation (AIUD) processes for every LEP patient identified as needing services, we hypothesized that improving quality of communication and interpretation would contribute toward decreasing LEP 48-hour RV. While improving LEP RV requires a complex approach including factors such as communication, discharge process and access to care, we began by addressing gaps in AIUD.

## METHODS

### Study Design and Setting

**Fig. 1.**
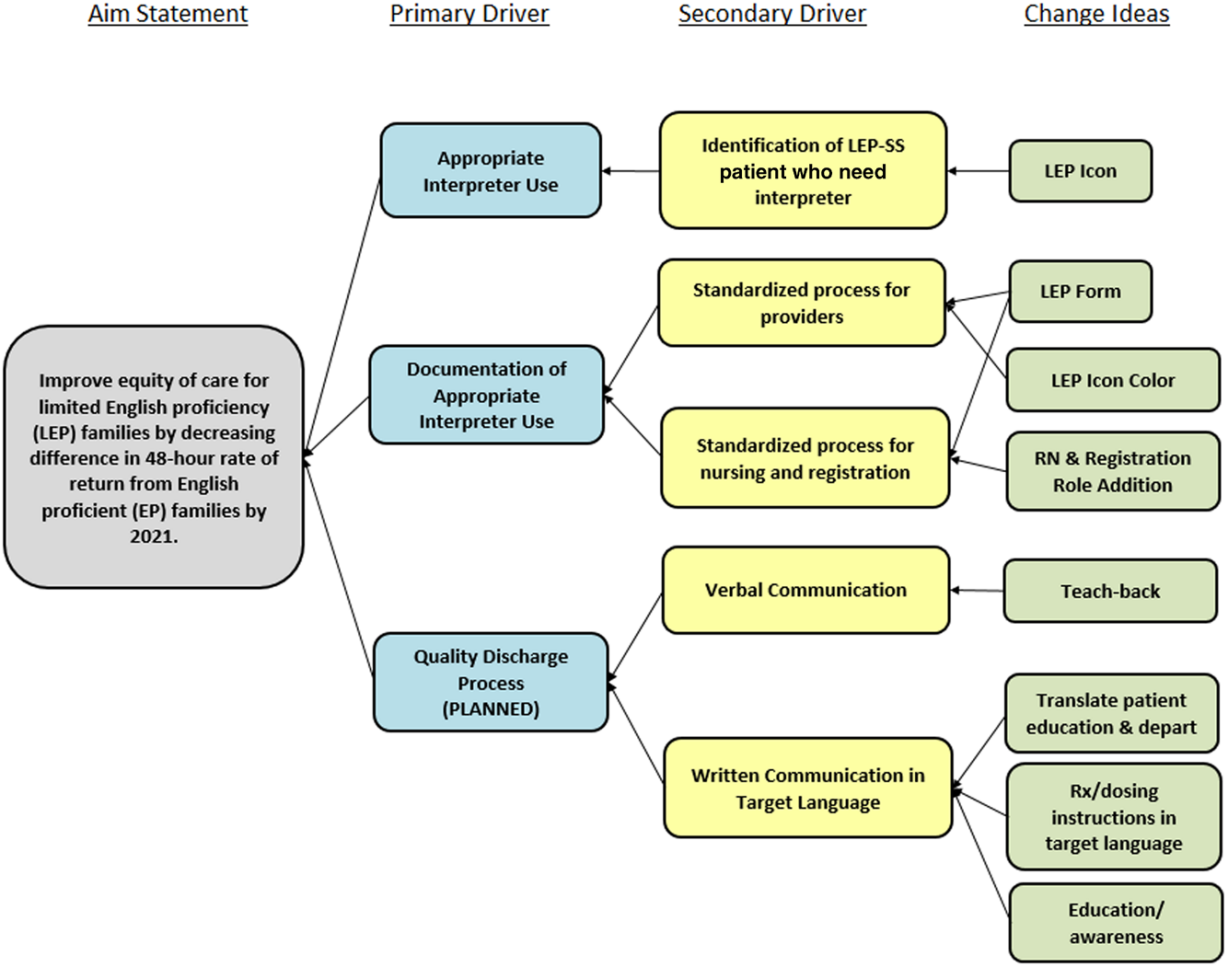
Key driver diagram showing the project aim, and the primary and secondary drivers that contribute toward achieving the aim.

This project occurred between October 2017 and October 2020 with several interval tests of change performed in the ED of a freestanding, urban, academic children’s hospital designated as a Level-1 Pediatric Trauma Center with an annual volume of approximately 53,000 patients. The hospital uses Cerner (Cerner Corporation, Kansas City, Mo.) as its primary electronic health record (EHR) for clinical documentation and Eclipsys (Atlanta, Ga.) for admit, discharge, and transfer. The Model for Improvement framework was used to evaluate existing processes, identify opportunities for improvement, and test and implement changes.^17^ A multi-disciplinary team developed a key driver diagram to guide the project (Fig. 1). Institutional Review Board approval was obtained for this project; it did not constitute human subjects research and was deemed a quality improvement project.

Before establishing a baseline, we reviewed 48-hour LEP RV (10/2017-04/2018), identifying and categorizing them as returning for the same or a different complaint.

The patient populations selected were divided into 2 groups: Spanish-speaking LEP (LEP-SS) patients and EP patients. For the LEP-SS group, data were collected on patients who designated Spanish as their primary language upon arrival to the ED or Urgent Care Center (UCC) and excluded from the population if their specified primary language was not Spanish. LEP-SS patients were chosen as the primary LEP population because this group represented the majority of the hospital’s ED LEP population (91.7%) (Table 1). The LEP-SS group was used to test changes that could later be applied to all LEP groups. Second, comparison group data were collected and analyzed on patients who designated English as their primary language upon arrival to the ED during the registration process. Of the LEP-SS group, a portion of these patients waived an interpreter, who were included in the overall analysis but not included in AIUD.

**Table 1.** Differences in Patient Characteristics in the Baseline, Postintervention, and Cumulative Study Periods

Patient Characteristics	Baseline N (%) (10/2017–05/2018)	PDSA1 and 2 Time Period N (%) (6/2018–02/2020)	PDSA3 Time Period N (%) 3/2020–10/2020	Cumulative Total N (%) (10/2017–10/2020)
ED patients*	36,419	96,114	21,534	154,067
Total EP ED patients	35,431 (97.3)	92,825 (96.6)	20,824 (96.7)	149,080 (96.8)
LEP-SS ED patients	910 (2.5)	3001 (3.1) 2755†	662 (3.1)	4573 (3.0) (91.7)‡
Total LEP ED patients	988 (2.7)	3289 (3.4)	710 (3.3)	4987 (3.2)
LEP-SS waived interpreter	15 (1.6)	129 (4.3)	36 (5.4)	180 (3.9)
LEP-SS AIUD	325 (35.7)	1601 (53.3)	427 (64.5)	2353 (51.4)
LEP Icon was Activated§	NA	1766 (64.1)	458 (69.2)	2224 (65.1)
Mean ED LEP LOS (min)	175.3	190.4	181.0	186.1
Mean LEP VRI encounter (min)	11.9	15.8	23.4	16.5
Interpreter modes				
VRI	278 (86.9)	1267 (79.3)	392 (91.6)	1937 (82.6)
LSI	3 (0.9)	183 (11.5)	3 (0.7)	189 (8.1)
Hospital-approved medical interpreter	24 (7.5)	111 (7.0)	26 (6.1)	161 (6.9)
OPI	15 (4.7)	35 (2.2)	7 (1.6)	57 (2.4)
LEP 48-h return visits	30 (3.0)¶	96 (2.9)	26 (3.7)∥	152 (3.1)
EP 48-h return visits	978 (2.8)¶	2482 (2.7)	479 (2.3)∥	3939 (2.6)

*Total ED patients do not include 153 patients who left primary language preference blank.

†Total LEP-SS ED patients after LEP icon activation only (9/2018–2/2020).

‡Percent of LEP-SS ED patients out of the Total ED LEP patients (4573/4987).

§Percentages for this row calculated based on total LEP-SS patients only for time periods where LEP icon was present (9/2018–10/2020) (cumulative calculation 2224/3417).

¶Baseline *P* < 0.001 comparing LEP and EP 48-h return visits.

∥After all interventions (PDSA3 Time Period) *P* < 0.001 comparing LEP and EP 48-h return visits.

LEP-SS baseline data were collected between 10/2/2017 and 5/7/2018 to evaluate patient identification, interpreter use, documentation, and 48-hour return visits to the ED. Monthly data collection was set for 30 days postdischarge of admitted patients. Additionally, we collected data further defining the type of interpreter used. As balancing measures, ED LOS and average video remote interpreter (VRI) encounter time were monitored for the overall LEP ED population. To represent the UCC LEP-SS population, comparable baseline data were collected from 1 UCC location with the highest volume of LEP-SS patients. UCC data were collected and analyzed separately from ED data.

The project team convened ED providers (physicians and nurse practitioners), pediatric residency, nursing, registration, information services, patient experience, and language services. After conducting interviews and direct observations, the project team developed a process map of the current ED/UCC state to identify LEP patients and provide appropriate interpretive services. No standard processes were found for ED LEP patient identification, communication regarding interpreter need to clinical and nonclinical staff, or provider documentation of appropriate interpreter use. The team then identified areas of opportunity for an improved future state.

### Interventions

To address the gap in identification and improve ED clinical and nonclinical staff situational awareness of patients’ LEP status, an LEP icon was designed (PDSA1: 9/18/2018 to 7/30/2019) to appear on the ED electronic tracking board. If a patient or family needed an interpreter during the ED triage process, the triage nurse selected “Language” as one of the “Barriers to Care” in the EHR triage form. This resulted in the appearance of the LEP icon in the “Events” column on the tracking board for that particular patient. This icon remained visible to all providers, nurses and registration staff. The icon consisted of the abbreviation “LEP” in black, capitalized font. Any other hospital staff member with access to Cerner EHR could also manually activate the LEP icon at any time during the visit if they determined an interpreter was needed or if the family later requested one.

A secondary gap identified inconsistencies and low compliance in the documentation of interpreter use. A new “LEP Form” (PDSA2: 7/31/2019 to 4/6/2020) was linked to a quick access button in the EHR, which facilitated real-time standardized documentation of interpreter service provision. This form was designed to require only 3 clicks to complete (approximately 15 seconds) and replaced free text in provider notes as interpreter use documentation. The LEP Form recorded interpreter use, the type of interpretive services used, waiver of a professional medical interpreter, and the reason for the waiver, if applicable.

To further improve compliance and consistency across roles, visual modifications to the documentation workflow were made (PDSA3: 4/7/2020 to 10/31/2020). The LEP icon’s initial color was changed from black to red font, and nursing and registration staff were asked to document interpreter use on the LEP Form in the same way as providers. Upon completion of the LEP Form by an ED provider, the icon turned to a green font. The utilization of the LEP Form by nursing and registration did not change the icon color to green font in an effort to ensure that ED providers were consistently documenting interpreter use. The project team also extrapolated and systematically implemented the ED LEP workflow, including the LEP icon and form, in the health system’s 4 urgent care centers (UCCs). The UCCs have an almost identical workflow compared with the ED workflow; therefore, it was the next logical step to spread to these locations.

### Measurements and Outcomes

Because all patients who need an interpreter should receive one, we determined the overall AIUD goal should be 100%. Utilizing a monthly electronic report of all ED and UCC LEP-SS patients, the project team completed manual reviews on each LEP-SS chart to evaluate AIUD. AIUD during PDSA cycles 1–3 was confirmed by the presence of either a free text notation in the provider’s EHR note or completion of the LEP Form. An appropriate interpreter was defined as one of the following: an in-person language services interpreter (LSI), a video remote interpreter (VRI), a staff member certified by the hospital as a Spanish medical interpreter (HMI), or an over-the-phone interpreter (OPI). If a LEP-SS patient/family declined a professional interpreter, we excluded them from the AIUD rate because an appropriate interpreter was not used. During PDSA3, UCC AIUD was measured for LEP-SS patients at the location with the highest census of this population.

Our primary outcome measure was 48-hour RV for LEP and EP populations, process measures included AIUD and LEP icon activation, and the balancing measures were ED LOS and VRI encounter time. All data were analyzed using statistical process control (SPC) charts created using Minitab Statistical Software v.19 (Minitab, Inc., State College, Pa.). Special cause was determined by a single point outside the control limits, while centerline shift was determined by a run of 8 or more points in a row above (or below) the centerline.^18^ Data were analyzed before and after all interventions, using the 2-proportions test, and statistical significance was determined when *P* < 0.05.

## RESULTS

Table 1 displays the patient characteristics of the baseline and postintervention (PDSAs1-3) periods. Of the total 154,067 ED patients, 4573 (3.0%) were in the LEP-SS group, with 180 (3.9%) waiving a professional interpreter, and 149,080 (96.8%) EP patients were in the comparison group.

**Fig. 2.**
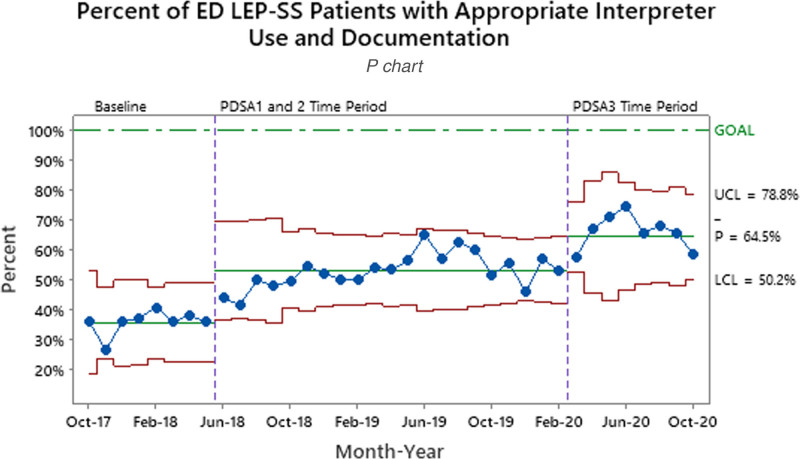
Percent of LEP-SS patients who had an interpreter used and documented in the ED. LEP-SS patients who refused or did not need an interpreter were not included. Process measure tests are performed with unequal sample sizes.

**Fig. 3.**
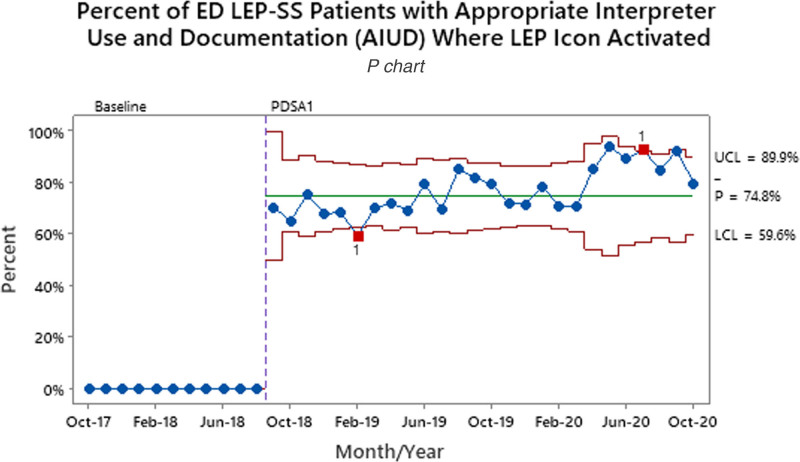
ED LEP-SS patients who had the LEP icon activated for identification and awareness along with AIUD. The red squares at Feb-19 and Jul-20 indicate special cause points outside control limits. LEP-SS patients who refused or did not need an interpreter were not included. Process measure tests are performed with unequal sample sizes.

**Fig. 4.**
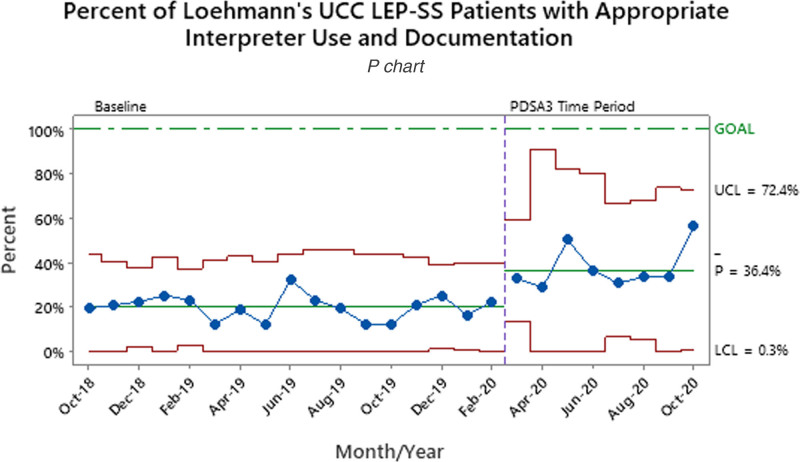
Percent of LEP-SS patients who had an interpreter used and documented in the UCC location with the most baseline LEP visits.

Patients waiving an interpreter increased from 1.6% to 5.4% between the baseline and final PDSA periods (Table 1). During the baseline period, 35.7% of ED LEP-SS patients had AIUD. After the implementation of LEP icon color changes and role additions (PDSA3), the percent of patients with AIUD increased to 64.5% (Fig. 2). Overall, LEP icon use increased by 10.4% between the PDSA1/2 and PDSA3 time periods. Overall, LEP icon use increased from 58.8% to 69.2%. For ED LEP-SS patients with the LEP icon activated and used an interpreter, AIUD increased to 74.8% after initial implementation (Fig. 3). The UCC LEP-SS patients with AIUD increased from a baseline of 19.8% to 36.4% after going live to all UCCs with LEP workflow (Fig. 4).

**Fig. 5.**
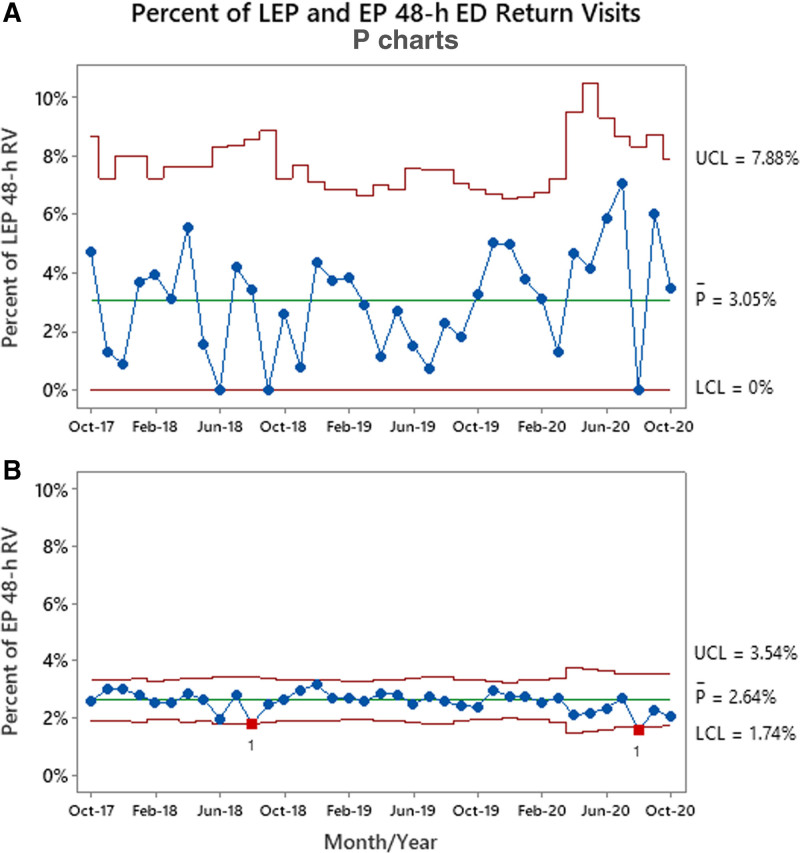
Percent of LEP ED and EP ED patients who returned to the ED within 48 hour of initial visit. A, B, Percent of LEP ED and EP ED patients who returned to the ED within 48 hours of initial visit. The red squares at Aug-18 and Aug-20 indicate special cause points outside control limits. Outcome measure tests are performed with unequal sample sizes.

Of the 4987 total LEP patients, 152 (3.1%) had a second ED visit within 48 hours (Fig. 5a). Of the total 149,080 EP patients in the comparison group, 3,939 (2.6%) returned within 48-hours (Fig. 5b). Pre-PDSA1, when comparing LEP and EP populations, the RV rate yielded a *P* value of < 0.001, and after all interventions the *P* value remained at < 0.001. Balancing measures for all ED LEP patients saw no significant changes where ED LOS and VRI encounter time averaged 186.1 and 16.5 minutes, respectively (Table 1).

## DISCUSSION

The timely identification of LEP patients can help with the proper delivery of the right care at the right place and right time. Ensuring equity of care through identifying language needs and using and documenting professional interpreters throughout the ED visit can have a profoundly positive impact on patient outcomes.^1,15,16^ Other studies have shown the LEP population to be at a higher risk for return visits with admission compared with EP patients as well as an association between Spanish language and increased RVs.^11,14^ Our project was different in that we sought to decrease LEP RVs through early LEP identification and professional interpreter use. The standardization of LEP identification and AIUD gave ED/UCC staff a heightened awareness of LEP needs, and provided direction and a way to measure processes to ensure equity of care. We believe system adoption of this project, its related interventions, and transparent data positively impacted organizational culture and helped further advance discussion regarding health care disparities. This was evidenced by increased AIUD rates, staff reportedly seeking out information on how to better serve the LEP population with greater frequency and feedback from ED staff that LEP workflow and concerns are now part of a daily discussion. Problem awareness is often enough to provide the spark for future change.

During the project’s initiation, where situational awareness was increased due to key stakeholder involvement, the Hawthorne effect was appreciated, as shown by a rise in AIUD before implementation of any process changes.^19^ Despite the implementation of standardized documentation using the LEP Form in PDSA2, the expected increase in AIUD was not appreciated until after PDSA3. Some factors potentially affecting this delay were staffing shortages of both LSI and ED nursing staff, combined with an acute rise in ED census. We saw LSI use significantly increase between 6/2018 and 2/2020 from its baseline rate of 0.9% to 11.5% (*P* < 0.001) (Table 1). This was due to the organization employing an on-site evening-shift Spanish interpreter from 2/2019 to 8/2019, increasing available interpreters to 3. This new evening availability coincided with hours of increased ED census. Unfortunately, as of 8/2019, staffing decreased to 1 interpreter. Nonetheless, this resource was widely utilized and preferred by both staff and families. The organization is actively recruiting Spanish interpreters to reach optimal staffing levels.

The LEP Form and icon changes implemented in PDSA3 were done to encourage provider accountability and situational awareness (especially during transitions in care) and meet the hospital policy requirements for documentation of services provided. The improvements noted during PDSA3 may be due to multiple factors, including improved situational awareness, addition of nursing and registration roles to documentation, decrease in the census, and acquisition of a LSI. Spread to UCCs also may have provided consistency in the workflow for staff between the outpatient and ED settings when LEP patients transfer between sites.

The UCC location with the most LEP visits was used as a barometer for all 4 UCCs to measure success and spread to other UCC locations. Though the LEP-SS population was used as our test group, all interventions were applicable to all LEP groups to ensure our goal of using the knowledge gained from these tests of change to drive change with the overall LEP population in the ED and UCCs.

The significant increase in AIUD throughout multiple PDSA cycles suggests either an increase in interpreter use, documentation of interpreter use, or both. Standardization of identification and documentation processes contributed to these improvements, yet the 48-hour RV rate for LEP patients remained stable and did not decrease in significance (*P* > 0.05) as we hoped. We noted a wider variation in LEP RV (0%–7%) compared with EP RV rates (1.5%–3.2%). This may be due to the smaller LEP population as well as demonstrating the complexity of the problem. Though professional interpreter use improves patient outcomes such as return visits, by itself, it had no significant effect. One possible reason these improvements did not affect the RV rate could be that the actual provision of interpretive services was already occurring most of the time, but was inconsistently documented. Before this project, there was no process for collecting and analyzing data to understand our baseline. This project provided the opportunity to create reliable data through data collection and process improvement. With the addition of standardized processes, we can gauge a real sense of baseline interpreter use and documentation and use that reliable data to more precisely target and improve the outcome of 48-hour LEP RVs. The improvements could have brought awareness to this patient population and improved the consistency of interpreter use, though not enough to affect the RV rate significantly. It is more likely that the improvements relate more to documentation than to an increase in interpreter use itself. This increase in documentation would also account for the rise in waiver of interpretive services.

## LIMITATIONS

There are several limitations in our project. To determine our baseline interpreter use, a time-intensive and thorough manual chart review was performed. Documentation of interpreter use was inconsistent and may have resulted in missing patients where interpreters were used. The ability of a patient or family to decline professional interpreter services limits our ability to keep the LEP-SS population homogenous. Additionally, although the LEP icon was a tremendous advancement for our health system, an icon cannot ensure that an interpreter will be used. AIUD was measured by evidence of documentation, noted in the provider note as free text or in the LEP Form, which does not ensure an interpreter was used for every conversation throughout the visit. To obtain a more accurate measurement of the quality and consistency of interpreter use, the number of times an interpreter was used and the purpose of each encounter such as triage, medical exam, and discharge, should be considered for data collection. By assessing the compliance of all roles using an interpreter throughout a visit, we could determine the visit’s overall quality instead of using one-time interpreter use.

Currently, the LEP icon is available for use in the ED and UCCs and is connected with the Cerner EHR; however it is visit-specific and does not follow the patient throughout the health system once activated. Therefore, staff (clinical and nonclinical) rely on the activation of the LEP icon during each visit to communicate patient needs. Another limitation is that the use of 1 UCC as a representative may not provide an accurate picture of AIUD for all UCCs.

### Future Directions

Future interventions will address education and other factors that may affect LEP RV. A web-based training module focusing on culturally effective healthcare practices, including working with LEP patients, has been developed and will be implemented and completed annually by both clinical and nonclinical audiences across our health system. The team will focus on the planned primary driver “quality discharge process” and related components such as assessing caregiver comprehension, availability and use of translated documents, primary care visits, and socioeconomic factors that may affect patient outcomes. In addition, finding a way for the LEP identification to follow patients throughout the health system, instead of only being specific to the ED or UCCs, would help reduce identification errors and ensure efficiency in providing language services.

## CONCLUSIONS

Instead of attempting to decrease LEP RVs through increased interpreter use alone, the factors that contribute to the 48-hour RV rate may be multi-factorial and require a more holistic approach. In the future, other factors such as cultural competency and interpreter use education, access to care issues, and standardized discharge processes that address caregiver comprehension should be considered.^15,20^ To ensure families are better equipped to care for their children at home and prevent unnecessary ED RV, it may be more effective to focus on how all ED/UCC encounter components fit together to affect patient outcomes instead of interpreter use by itself.

In conclusion, although our data showed improvement in interpreter use and documentation, this alone did not yield improvements in return visits. A more holistic approach is required to have a more substantial effect on LEP 48-hour RVs.

## DISCLOSURE

The authors have no financial interest to declare in relation to the content of this article.

## ACKNOWLEDGEMENTS

Marti Bevan, BBA, MSN, RN, RN-BC was integral in providing feedback and creating and implementing tests of change throughout the project related to nursing informatics.
